# Presence of higenamine in beetroot containing ‘foodstuffs’ and the implication for WADA‐relevant anti‐doping testing

**DOI:** 10.1002/dta.3383

**Published:** 2022-10-25

**Authors:** Amy E. Leaney, Jenna Heath, Emma Midforth, Paul Beck, Paul Brown, Deborah H. Mawson

**Affiliations:** ^1^ LGC Ltd Fordham Ely UK

**Keywords:** beetroot, higenamine, supplement, WADA

## Abstract

Higenamine is an alkaloid found within plant species including some that are used in traditional Asian and Chinese herbal medicines. Identified as having mixed mode adrenergic receptor activity, higenamine is present within some nutritional supplements marketed for stimulant and/or weight loss. Its inclusion within nutritional supplements can be via its natural presence within botanical ingredients or as a synthetic additive, often added in mg amounts. The World Anti‐doping Agency (WADA) prohibited list has contained higenamine since 2017 as banned at all times in the beta‐2 agonist (S3) category, with a reporting level of 10 ng/ml for the free parent form in urine. In this study, an investigation into the content of beetroot or beetroot‐containing foodstuffs and supplement products was conducted. Higenamine was confirmed as present within the majority of foodstuffs and supplements, with experimental evidence that higenamine can arise within beetroot extracts through heating. The results in this paper demonstrate the first reported evidence of a link between beetroot and this WADA prohibited substance. To investigate the link between intake and excretion, concentrated beetroot drinks were consumed by six individuals and higenamine quantified in their urine. Free higenamine was detected in the urine of all individuals, with maximum measured concentration in samples of less than 1% of the current WADA reporting limit. Although the risk of an inadvertent doping violation by consumption of the foodstuffs and products investigated in this study is low, beetroot as a source of higenamine should be considered by athletes.

## Graphical abstract

An investigation into the presence of higenamine in beetroot‐based foodstuffs and the implications for WADA‐relevant anti‐doping testing. Higenamine has been detected in beetroot‐based foodstuffs. A feeding study has been conducted and the free analyte levels found in urine were extremely low compared with the WADA reporting limit.

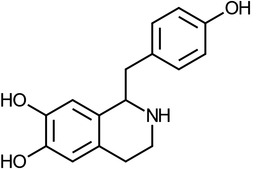

## 1 INTRODUCTION

Nutritional supplements rich in nitrates are often utilised by both amateur and elite athletes for a range of health and performance benefits. For example, dietary nitrates have been linked with cardio‐protective and beneficial vascular effects,^1^, ^2^, ^3^ and products containing these compounds are often sold as ‘pre‐workout’ supplements. Notably, beetroot‐based supplements have grown in popularity over the last decade due to their naturally high dietary nitrate content.^3^

The inclusion of botanical ingredients in supplements can present additional challenges and concerns to a consumer from both a health and a doping control perspective, particularly when their bioactive content and associated levels aren't known. The World Anti‐Doping Agency (WADA) prohibited list^4^ provides a comprehensive summary of substances and methods prohibited in sport. Included within the list are compounds, which could potentially be introduced into a supplement via use of a natural extract; examples include compounds such as octopamine, which can be found in bitter orange, and higenamine.

**FIGURE 1:**
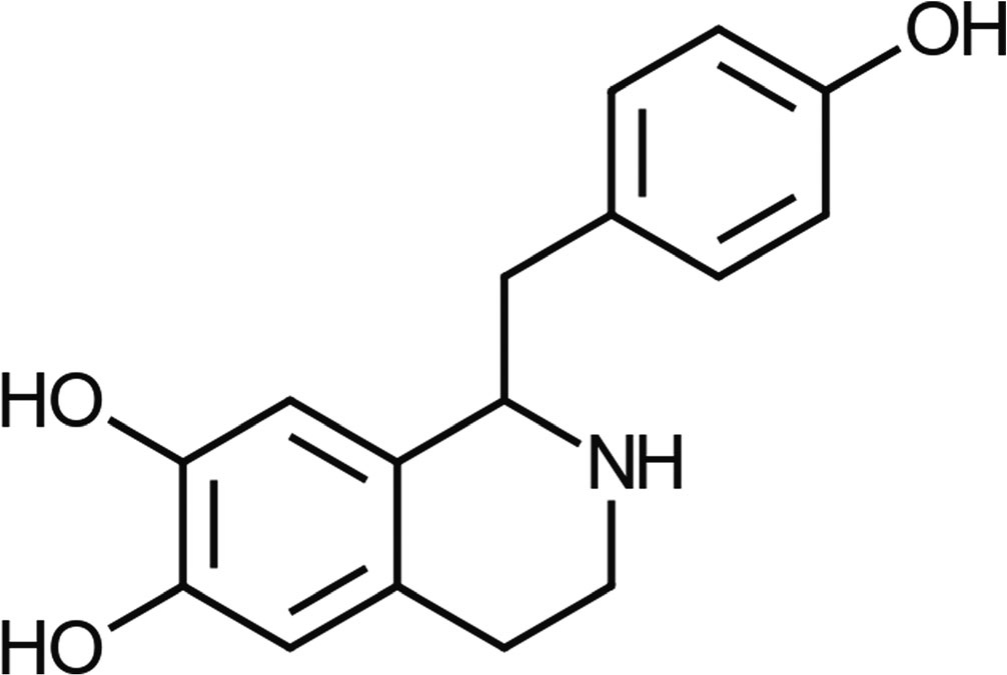
Chemical structure of higenamine

Higenamine [(±)‐higenamine, norcoclaurine, 1‐(4‐hydroxybenzyl)‐1,2,3,4‐tetrahydro‐6,7‐isoquinolinediol] (Figure 1) is an alkaloid found within numerous plant species, including *Annona squamosa*, *Aconitum carmichaelii* and *Plumula nelumbinis*,^5^, ^6^, ^7^ some of which are commonly used within Asian and Chinese herbal medicines.^8^, ^9^, ^10^ Identified as having mixed adrenergic receptor activity, clinical data relating to safety and efficacy of higenamine are extremely limited and the health risks of consumption are not currently understood.^9^, ^11^ Higenamine is present in some nutritional supplements; products which are typically marketed for their stimulant/weight management properties. Higenamine may appear on product label declarations either as higenamine, its chemical name, or its inclusion may be inferred by reference to a botanical ingredient. In some cases where a botanical source is listed, the presence of higenamine is not always from natural sources, but may be chemically synthesised and present in higher amounts than would be anticipated given the botanical source.^12^, ^13^

Higenamine has been prohibited for use both in and out of competition by WADA under section S3 (beta‐2 agonists) of the prohibited list since 2017.^14^ Adverse analytical findings (AAFs) for higenamine in doping control samples pre‐date its explicit inclusion on the prohibited list, with 55 occurrences reported for 2016.^15^ Since its inclusion, higenamine continues to account for a large proportion of findings in the S3 category—accounting for 30% of AAFs (46 occurrences) in 2019.^16^ Current guidelines^17^ state that an adverse finding for higenamine will be reported where the minimum reporting level (in respect of the free parent compound) is found to be >10 ng/ml in urine.

During recent testing of supplement samples by the authors, the presence of higenamine was detected in multiple samples containing beetroot material as the only listed botanical ingredient. Its presence was unexpected due to the absence of information supporting the natural presence of higenamine within the Betoideae and wider Amaranthaceae family of plants. The study presented investigates the previously unreported presence of higenamine in beetroot containing foodstuffs and supplements and investigates the relationship between beetroot intake and excretion profiles from a WADA testing perspective. This information will help inform both athletes and the supplement industry, thereby helping to safeguard health, reputation, and the integrity of sport.

## 2 EXPERIMENTAL

### 2.1 Reference standards, chemicals, reagents and beetroot products

Reference standards for higenamine and higenamine‐D_4_ (internal standard: IS) were obtained from Toronto Research Chemicals (Toronto, Canada) and BDG synthesis (Wellington, New Zealand) respectively. Bond Elut NEXUS solid phase extraction (SPE) cartridges and Oasis HLB SPE plates (10 mg) were purchased from Agilent (Stockport, UK) and Waters (Elstree, UK), respectively. All other solvents and analytical reagents were purchased from Fisher Scientific (Loughborough, UK) or Sigma Aldrich (Gillingham, UK). Beetroot foodstuffs were sourced from a UK supermarket and other commercial suppliers. Where possible, foodstuffs were selected where beetroot or beetroot extract represented the only or the major botanical ingredient.

### 2.2 Analysis of fresh and processed beetroot products

#### 2.2.1 Sample preparation and extraction

Samples of purchased foodstuffs were prepared as follows: raw beetroot was homogenised using a domestic juicer to obtain in excess of 100 ml of liquid. Cooked and pickled beetroot products were chopped and homogenised to produce a pulp. Liquid‐ and powder‐based products were invert mixed prior to sampling and for tablets, several were ground to a fine powder which was weighed.

Quantification of higenamine in the beetroot products was achieved via a standard addition approach. Aliquots of each product were fortified using 50–1000 ng/g higenamine to construct an eight point calibration line in each product. All samples were augmented with IS (100 ng) and then extracted by the addition of methanol followed by SPE using Bond Elut NEXUS cartridges. After addition of 2.5 M ammonium acetate pH 6.8, samples were loaded on to a pre‐conditioned cartridge, washed with 10% methanol in water and eluted using 2% formic acid in acetonitrile. Following evaporation to dryness, samples were reconstituted in methanol: 0.1% formic acid in water, 5:95 (v:v).

To aid in the determination of the source of higenamine, aliquots of beetroot juice obtained from homogenisation of raw beetroot were incubated under different conditions; one at room temperature for 5 h, one at 80°C for 1 h and the third at 80°C for 5 h. Following incubation, samples were left to cool to room temperature prior to the addition of IS and subsequent extraction using SPE.

#### 2.2.2 Liquid chromatography–high resolution accurate mass–mass spectrometry (LC‐HRAM‐MS)

Analysis was conducted using a Vanquish LC system coupled to a Q‐Exactive Focus Orbitrap mass spectrometer (Thermo, Hemel Hempstead, UK). The LC was equipped with a Waters X‐Select HSS T3 C18 column (2.1 × 75 mm, 2.5 μm) using 0.1% formic acid in water (A) and 0.1% formic acid in methanol (B) as mobile phases. A gradient was utilised, with flow rate 0.48 ml/min, starting at 0% B for 1 min increasing to 10% B within 2.2 min, 60% within 4 min and increasing to 100% B within 6.5 min. The system was held at 100% B for 1 min prior to re‐equilibration at starting conditions.

The mass spectrometer was operated in positive ionisation mode with a HESI‐II (Heated Electro Spray Ionisation) probe using a vaporiser temperature of 420°C and ion transfer tube temperature of 320°C. The spray voltage was 2500 V. The mass spectrometer was operated in full scan data dependent Discovery (ddDiscMS/MS) mode to capture both MS and MS^2^ data concurrently. MS^2^ data were derived from the fragmentation of ions detected above a threshold. Subsequent analysis was performed utilising Parallel Reaction Monitoring (PRM) mode to ensure MS^2^ data were captured for both higenamine and the IS. Data were processed using Thermo XCalibur version 4.0.27.21 QualBrowser and Thermo TraceFinder version 4.1.

### 2.3 Urinary excretion study

#### 2.3.1 Sample preparation and extraction

Solutions of higenamine were diluted into blank human urine to give calibration standards and quality control (QC) samples over the range 50–20,000 pg/ml. Higenamine was extracted from human urine using Waters Oasis HLB SPE plates (10 mg). Urine samples were prepared for SPE by the addition of IS and acidification with 1% formic acid aqueous.

The SPE plate was conditioned and equilibrated using sequential additions of methanol and 1% formic acid aqueous prior to loading the sample. Following sample loading, the SPE cartridges were washed by stepwise additions of methanol: water (5:95, v:v), methanol: ammonia: water (5:5:90, v:v:v) and methanol: water (5:95, v:v). Higenamine was eluted from the SPE plate with methanol: 1% formic acid in water (7:3, v:v). The eluents were evaporated to dryness with oxygen‐free nitrogen at ambient temperature before being reconstituted using methanol: 0.2% formic acid in water (15:85, v:v).

#### 2.3.2 Liquid chromatography–tandem mass spectrometry (LC‐MS/MS) analysis

Analysis was conducted using a Vanquish LC system, coupled with an Altis triple‐quadrupole mass spectrometer (Thermo, Hemel Hempstead, UK). The mobile phases and column were as detailed above for HRAM analysis, however a different solvent gradient programme was employed: Chromatographic conditions were held with a flow rate of 0.5 ml/min at 98% A for the first 0.5 min, increased to 25% B over the next 3.5 min and then increased to 95% B and the conditions held until 5 min, after which time the system was returned to starting conditions and re‐equilibrated.

The mass spectrometer was operated in positive electrospray ionisation mode with a vaporiser temperature of 400°C and ion transfer tube temperature of 350°C. The spray voltage was 3500 V. Collision energies were set at 26 eV for the fragmentation of higenamine and the deuterated IS, for the SRM transitions *m/z* 272 > 107 and 276 > 108 for higenamine and the IS, respectively. Calibration lines (analyte/IS peak area vs analyte concentration) were constructed using Thermo XCalibur version 4.0.27.21 QuanBrowser using a weighted linear regression.

#### 2.3.3 Method validation

The method was validated with reference to the Bioanalytical Method Validation Guidance for Industry^18^ and consisted of assessment of precision and accuracy, selectivity, stability in urine, recovery and matrix effects. All experiments performed within the stipulated criteria.

#### 2.3.4 Supplement administration study

Samples were collected from healthy volunteers at LGC Ltd (Fordham, UK) and used in accordance with informed consent and the in‐house bio‐ethics committee approval. Six working age adults (three male and three female), free from health conditions necessitating prescription medication, each consumed two characterised beetroot‐containing supplement drinks according to the manufacturer's recommended daily intake. Urine samples were collected by the individuals before consumption and then for up to approximately 24 h post dose. Samples were centrifuged at 3000 *g* for 10 min, the supernatant transferred to clean tubes and frozen at nominally −20°C until analysis.

## 3 RESULTS AND DISCUSSION

### 3.1 Identification and assessment of higenamine concentrations in beetroot foodstuffs

Initial investigations regarding the connection between higenamine and beetroot focused upon the analysis of a range of commercially available products. These included unprocessed foods (raw beetroot), processed products (e.g., pickled beetroot) and processed foods that had undergone significant transformation (e.g., concentrated beetroot).

**FIGURE 2:**
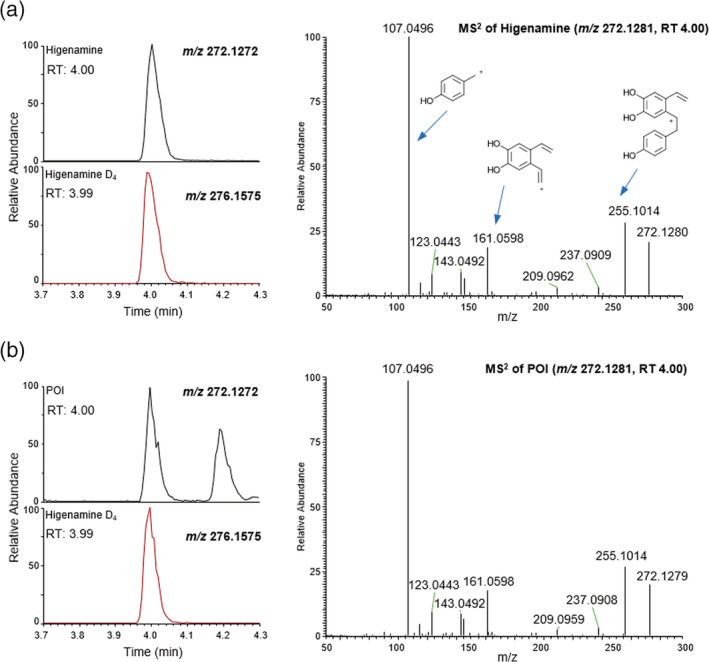
Analyte (top left) and internal marker (bottom left) ion chromatograms and analyte MS^2^ spectra (right) for (a) higenamine and (b) the peak of interest (POI) in an extracted commercially available concentrated beetroot juice. The likely structures of product ions evident in the MS^2^ spectra are shown for the higenamine reference standard. [Colour figure can be viewed at wileyonlinelibrary.com]

With the exception of the raw beetroot, the analysis of all other foodstuffs revealed evidence of the presence of higenamine. Identification was based upon the relative (to the internal standard) retention time of the peak of interest, accurate mass and isotope distribution of the intact precursor ion, and *m/z* values and relative intensities of product ions evident in the MS^2^ spectra. Data were compared directly to results generated from concurrent analysis of reference standards, and excellent concordance found in all cases (e.g., Figure 2) allowing for confidence in the identification of the analyte.

Higenamine concentrations were extrapolated via standard addition and are summarised in Table 1. An estimate of the total daily higenamine intake via consumption of the products is also provided for context of the dietary significance of levels. These estimates are based upon the maximum daily intake indicated by the manufacturer (where specified on product labels) or NHS single vegetable portion size recommendations where available.^19^

**TABLE 1:** Summary of the higenamine content measured in beetroot‐based foodstuffs

Reference number	Description of purchased product	Measured higenamine concentration	Recommended daily serving size*	Estimated higenamine in recommended daily serving
S1	Raw beetroot	0 ng/g	80 g	0.0 μg
S2	Cooked beetroot	38 ng/g	80 g	3.0 μg
S3	Pickled beetroot	37 ng/g	50 g	1.9 μg
S4	Beetroot juice drink	51 ng/g	200 ml	10.2 μg
S5	Beetroot concentrate drink	312 ng/g	35 ml	10.9 μg
S6	Beetroot powder food supplement	86 ng/g	5 g	0.4 μg
S7	Beetroot tablet food supplement	75 ng/g	1 tablet (0.84 g)	0.1 μg

* Based upon portion size recommended by the manufacturer (where applicable) or single adult portion size as recommended by the NHS.

No evidence of higenamine was found within the raw beetroot extract. Low levels of higenamine were detected in the cooked and pickled beetroot samples, with measured concentrations of 38 and 37 ng/g, respectively (S2 and S3; Table 1). The powder and tablet products contained relatively consistent levels of higenamine (86 and 75 ng/g, S6 and S7), with concentrations approximately double those detected within the cooked/pickled beetroot products. Within the two liquid foodstuffs (S4 and S5; Table 1), the levels observed are consistent with a concentration effect resulting in a higher amount of higenamine being present within the beetroot concentrate product than the juice (approximately 6 times greater). The maximum daily intake was found to be in the region of 10.9 μg following the consumption of 35 ml of beetroot concentrate. Notably, the levels of higenamine within the beetroot products investigated are much lower than those detected from other reported dietary sources. Lotus plumule, as well as supplements marketed as ‘pre‐workout’ or ‘fat‐burner’ can typically have concentrations several orders of magnitude higher (milligrams of higenamine per serving^12^, ^20^) than measured in the investigated beetroot‐based foodstuffs.

**FIGURE 3:**
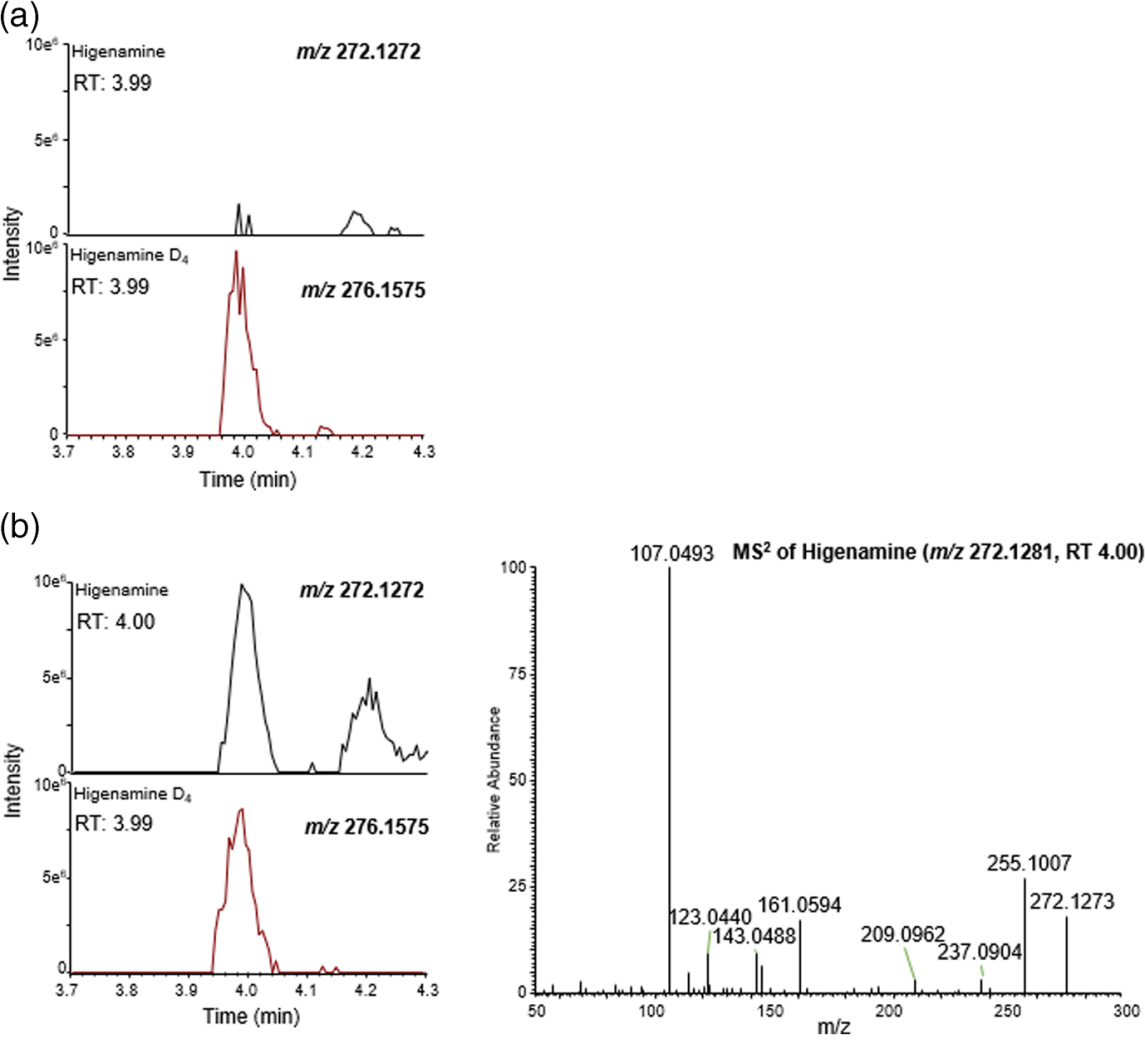
Analyte (top left) and internal marker (bottom left) ion chromatograms and analyte MS2 spectra (right, where shown) for raw beetroot extract (a) stored at room temperature for 5 h (nominally 18°C) and (b) following incubation for 5 h at 80°C. All ion chromatograms are presented to the same Y‐axis (intensity) scale. [Colour figure can be viewed at wileyonlinelibrary.com]

To aid in the determination of the source of the higenamine within the beetroot products, portions of homogenised raw beetroot (extracted juice) were subjected to different incubation temperatures (room temperature and 80°C) and durations, then analysed. Although negligible higenamine was found in room temperature samples, samples incubated at 80°C were found to contain higenamine, with the largest peak observed in the sample incubated for 5 h. Example chromatograms from this experiment are presented in Figure 3.

Beetroot contains high levels of betalains and polyphenols,^21^ which may represent possible precursors of higenamine, especially due to their propensity to degrade to smaller molecules when subjected to environmental factors (e.g., temperature, pH, exposure to light and enzymatic activities^22^). Although the mechanisms of higenamine formation within beetroot‐containing products is beyond the scope of this paper (particularly as the precise processes employed by manufacturers are not known by the authors), the concentrations within the different foodstuffs and presence in samples heated for prolonged periods would seem to suggest that there is a link between the degree of processing (particularly where temperature is a factor) and higenamine levels. Furthermore, any higenamine present in products where there is a significant technological processing may be concentrated further as part of manufacture through processes such as dehydration.

Regardless of the uncertainty regarding mechanism of formation, this paper represents the first reported evidence of a link between beetroot and higenamine in foodstuffs. As a WADA‐prohibited substance with a reporting level, it is important to understand the link between intake and excretion of higenamine; specifically in relation to beetroot products and the risk from a doping control perspective. A further study was conducted using a concentrated beetroot juice foodstuff to aid in the evaluation of this link.

### 3.2 Urinary excretion study

A method to quantify higenamine in human urine was developed bracketing the WADA reporting level for free higenamine (10 ng/ml) ranging from 50–20,000 pg/ml. In line with WADA guidelines,^17^ free higenamine only (i.e., non‐hydrolysed parent drug) was measured. Precision and accuracy were within criteria of ≤15% relative standard deviation and ± 15% relative error (20% criteria at the lower limit of quantification, LLOQ) at the tested concentrations. The QC performance is summarised in Table 2. Assessment of sensitivity and selectivity demonstrated a signal to noise ratio of at least five to one at the LLOQ for all batches, with no interferences observed for either the higenamine or IS transitions.

**TABLE 2:** Precision and accuracy data for the measurement of higenamine in human urine

	QC LLOQ	QC low	QC med	QC high
Nominal concentration (pg/ml)	50.0	150	2000	15,000
Mean concentration (pg/ml)	57.5	147	1970	15,300
Relative error (%)	15.0	−1.9	−1.3	2.2
Relative standard deviation (%)	2.7	4.7	1.9	1.9
*n*	5	6	6	6

The analytical method was used to measure free higenamine in human urine following consumption of two concentrated beetroot supplement drinks (on a single occasion as per the manufacturer's recommended maximum intake) by six individuals. The concentration of higenamine within the concentrated beetroot supplement drink was assessed prior to use, and the intake from the two drinks found to be 15.7 to 18.9 μg.

**FIGURE 4:**
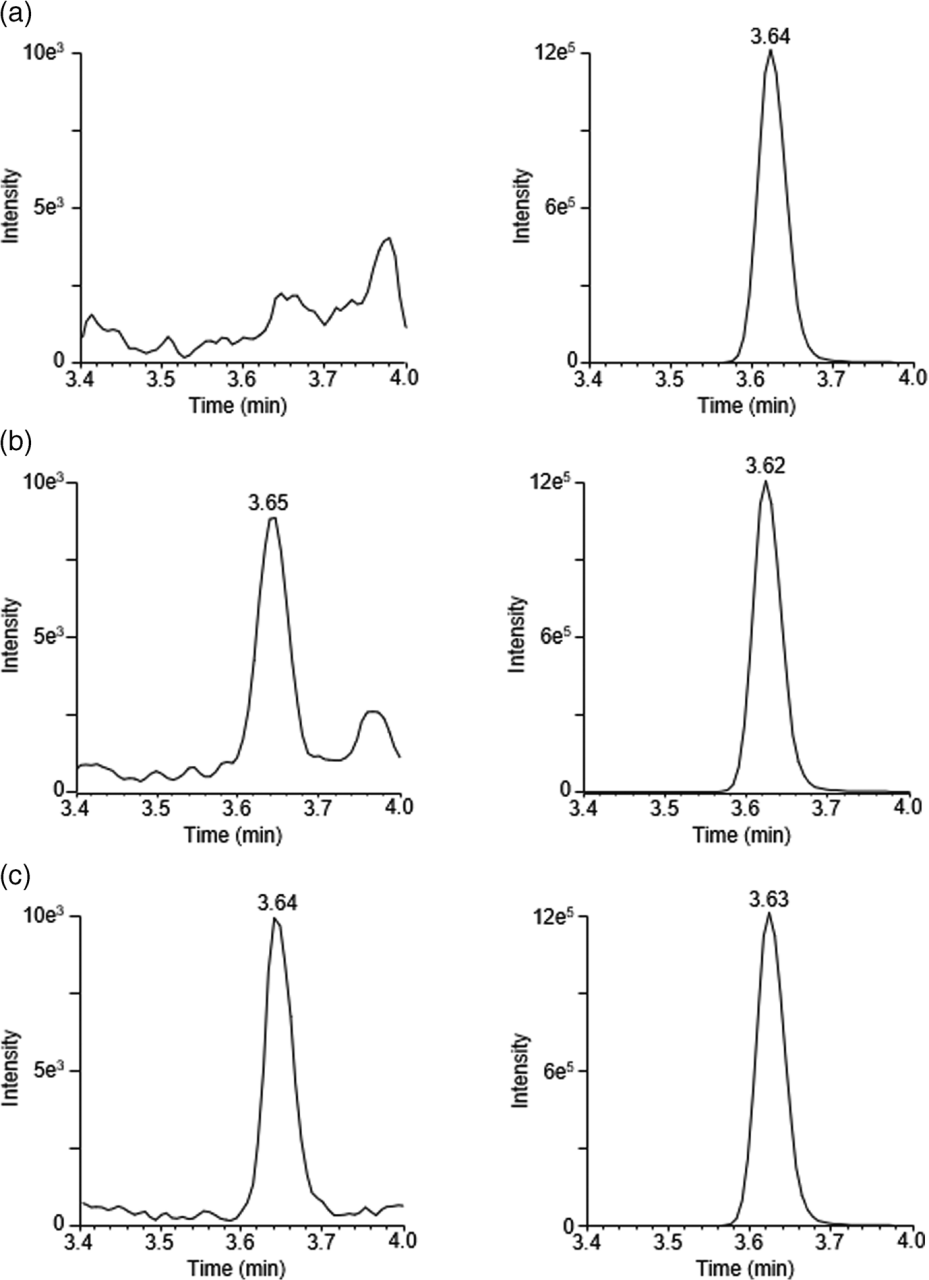
MRM chromatograms for higenamine (left) and higenamine‐D4 (right) in (a) a pre‐dose urine sample, (b) a post‐dose urine sample and (c) a urine LLOQ sample spiked with higenamine at 50 pg/ml. Data for all samples are presented with the same Y‐axis scaling.

Higenamine was not detected in urine samples from any of the volunteers prior to the consumption of beetroot‐based supplements. Following consumption, higenamine was detected in urine samples from all volunteers, with maximum concentrations for the different individuals and approximate excretion times reported in Table 3. The levels detected were far below the WADA reporting limit of 10 ng/ml, and in some individuals did not reach the assay LLOQ of 50 pg/ml, with a mean maximum urine concentration of 48.2 pg/ml measured approximately 4 h after supplement consumption. No differences were observed between the maximum concentrations measured in urine from males and females. Example chromatograms showing a pre‐dose urine sample, a urine sample post supplement consumption, and a spiked urine standard at the LLOQ are shown in Figure 4.

**TABLE 3:** Maximum urinary concentrations of higenamine measured and the time elapsed since the supplements were consumed

Individual	Nominal time (min)	Urine concentration (pg/ml)
Female 1	255	52.7
Female 2	320	66.3
Female 3	234	23.7 (BLQ)
Male 1	120	39.7 (BLQ)
Male 2	215	49.8 (BLQ)
Male 3	282	56.9

Abbreviation: BLQ, below the limit of quantification.

**FIGURE 5:**
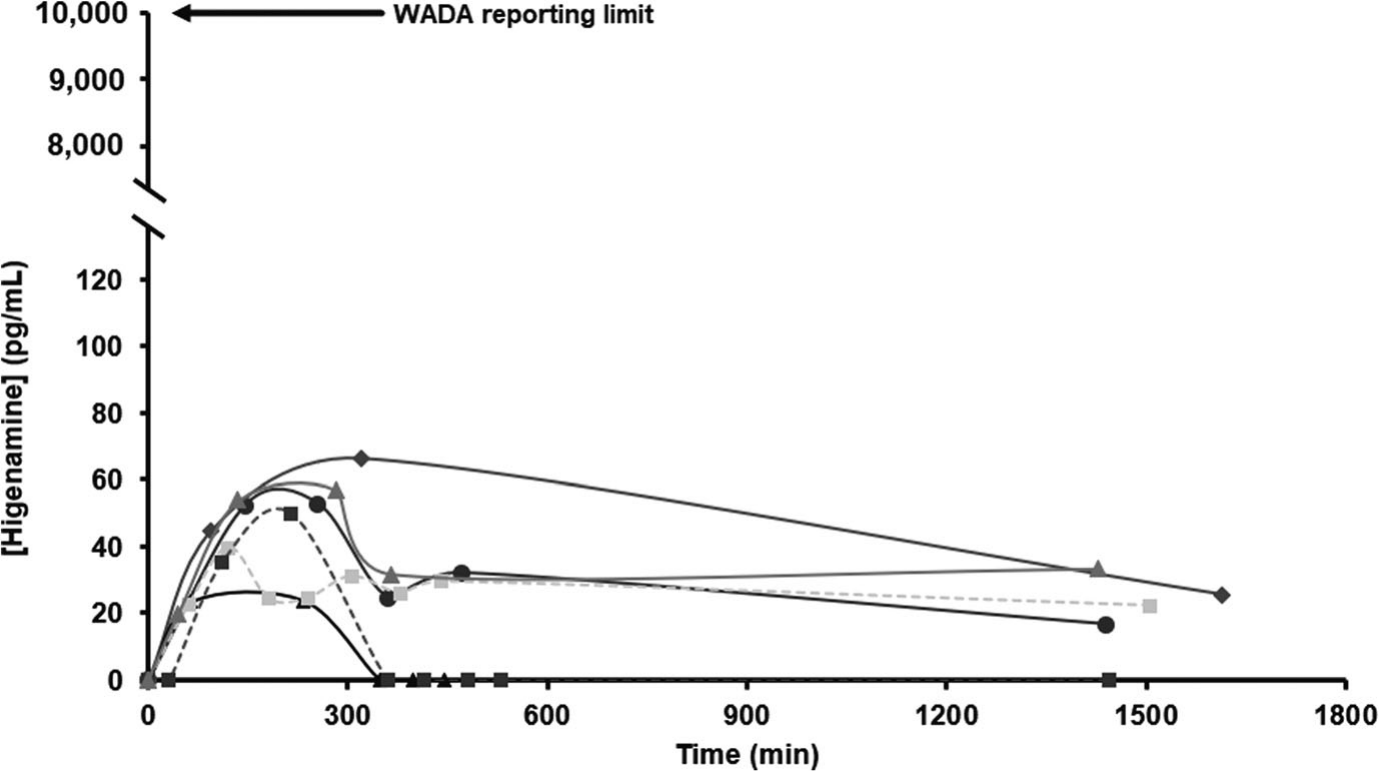
A graph of the concentrations of higenamine measured in human urine from six individuals following the consumption of concentrated beetroot shots

The excretion profiles of free higenamine measured in urine from the six individuals are displayed in Figure 5. Very low higenamine concentrations were detected following consumption of the concentrated beetroot supplement drinks, with maximum concentrations for all individuals reaching less than 1% of the WADA reporting level of 10 ng/ml. As reported previously, the half‐life of higenamine is approximately 8 min, and it is rapidly eliminated from the body.^12^, ^23^ Elimination is predominantly via phase II metabolism, with sulfate conjugates representing a major excreted form of higenamine.^5^, ^24^ Although not investigated here due to the focus of the study on WADA relevant (i.e., free, not total) levels, additional peaks are evident in the chromatograms of the post supplement samples that are absent from the pre‐dose samples and likely correspond to metabolites.

The supplements used for the excretion study had a recommended daily consumption of one or two drinks (shots) per day, with the recommended extended use of up to six consecutive days. Although this study was limited to consumption of two drinks on a single occasion, it is unlikely that the WADA threshold would be exceeded if the maximum recommended dosage and duration was followed. Furthermore, a recent study quantifying higenamine in human urine following the administration of lotus plumule three times daily for 3 days did not show significant bioaccumulation with repeated doses.^20^

The results generated as part of this study would suggest that the amount of higenamine consumed in any of the foodstuffs tested (following recommended intake) is unlikely to lead to excretion of WADA‐significant levels based upon the current reporting limit for free (i.e., non‐hydrolysed) higenamine in urine. Although the risk posed by higenamine derived solely from beetroot seems minimal, the onus is on athletes to ensure that any products, particularly those such as dietary supplements that are likely to have undergone significant processing or fortification, are from a reputable source and ideally subject to independent testing and evaluation.

## 4 CONCLUSION

This paper presents the first reported detection of higenamine within processed beetroot foodstuffs and evidence of formation of higenamine from a component within this vegetable. Different commercially available products have been tested and levels of higenamine are within the ng/g (ppb) range, with the maximum concentration in this study observed within a concentrated form of beetroot juice.

A subsequent administration study using concentrated beetroot drinks has been conducted. Following the manufacturer's recommended daily intake, low levels of free higenamine were observed in the urine samples of six volunteers. The concentrations detected were far below the current WADA reporting level for free higenamine in urine.

Consumption of beetroot‐containing foodstuffs, tested as part of this study, are unlikely to result in a doping violation (based on the current WADA guidelines/reporting levels) when consumed in line with manufacturer guidelines or recommended vegetable portion sizes. However, athletes should ensure that any products, particularly those such as dietary supplements that are likely to have undergone significant processing or fortification, are from a reputable source where products have been subject to independent testing and evaluation to assess risk.
